# Genome-Wide Identification and Characterization of the GRAS Gene Family in Lettuce Revealed That Silencing *LsGRAS13* Delayed Bolting

**DOI:** 10.3390/plants13101360

**Published:** 2024-05-14

**Authors:** Li Chen, Yong Qin, Shuangxi Fan

**Affiliations:** 1 College of Horticulture, Xinjiang Agricultural University, Urumqi 830052, China; 202040240001@bua.edu.cn (L.C.); xjndqinyong@126.com (Y.Q.); 2 Plant Science and Technology College, Beijing Vocational College of Agriculture, Beijing 102442, China

**Keywords:** lettuce, GRAS gene family, high-temperature stress, melatonin

## Abstract

Lettuce is susceptible to high-temperature stress during cultivation, leading to bolting and affecting yield. Plant-specific transcription factors, known as GRAS proteins, play a crucial role in regulating plant growth, development, and abiotic stress responses. In this study, the entire lettuce LsGRAS gene family was identified. The results show that 59 *LsGRAS* genes are unevenly distributed across the nine chromosomes. Additionally, all LsGRAS proteins showed 100% nuclear localization based on the predicted subcellular localization and were phylogenetically classified into nine conserved subfamilies. To investigate the expression profiles of these genes in lettuce, we analyzed the transcription levels of all 59 *LsGRAS* genes in the publicly available RNA-seq data under the high-temperature treatment conducted in the presence of exogenous melatonin. The findings indicate that the transcript levels of the *LsGRAS13* gene were higher on days 6, 9, 15, 18, and 27 under the high-temperature (35/30 °C) treatment with melatonin than on the same treatment days without melatonin. The functional studies demonstrate that silencing *LsGRAS13* accelerated bolting in lettuce. Furthermore, the paraffin sectioning results showed that flower bud differentiation in *LsGRAS13*-silenced plants occurred significantly faster than in control plants. In this study, the *LsGRAS* genes were annotated and analyzed, and the expression pattern of the *LsGRAS* gene following melatonin treatment under high-temperature conditions was explored. This exploration provides valuable information and identifies candidate genes associated with the response mechanism of lettuce plants high-temperature stress.

## 1. Introduction

Lettuce (*Lactuca sativa* L.) is a primary vegetable cultivated in fields or facilities, valued for its significant culinary and economic importance [1,2]. Unfortunately, during cultivation, it is susceptible to various abiotic and biotic stresses, including high salinity, drought, extreme temperatures (both high and low), and pathogen infections [3,4]. Particularly, it is prone to bolting under the influence of high temperatures, a process that limits the marketability of lettuce [5]. The entire life cycle of higher plants includes two stages: vegetative growth and reproductive growth. Flowering is a pivotal transition stage from the vegetative stage to the reproductive stage and is among the most crucial biological processes in plants. Bolting refers to the development of flowering stems on a crop before harvest, leading to seed production for propagation. Simultaneously, this process is regulated by the interaction of various environmental factors and endogenous developmental signals, ensuring that plants accumulate enough nutrients to reproduce offspring [6]. Significant research has been conducted to gain insight into lettuce plants’ responses to abiotic and biological stresses [7,8]. Many lettuce transcription factor (TF) families, such as WKRY [9], R2R3-MYB [10], GRF [11], and MADS [12], have been extensively researched. The GRAS family comprises genes encoding transcription factors, with the acronym ‘GRAS’ derived from the initials of gibberellic acid insensitive (GAI), a repressor of GAI (RGA), and scarecrow (SCR). This gene family is widespread in plants and plays a crucial role in plants’ responses to stress, growth, and development [13]. In *Arabidopsis*, mutations in the GAI and RGA domains within the GRAS gene family lead to semi-dominant gain-of-function mutations, resulting in the semi-dwarf phenotype [14]. The *RGL1* gene, acting as a negative regulator, controls various gibberellic acid (GA) responses, including flower development and stem elongation [15]. By studying the GRAS gene family in lettuce, we can gain a deeper understanding of the functions of these genes in the growth and development of lettuce and its response to stress. This understanding will enable us to design more effective lettuce breeding strategies aimed at improving adaptability and stress resistance of lettuce.

The GRAS proteins have been widely studied over the past decade [16,17]. These proteins consist of 400–770 amino acid residues and can be divided into variable amino (N-) terminal regions and highly conserved carboxyl (C-) terminal regions [18,19]. The N-terminus comprises chaotic domains involved in molecular recognition, exhibiting a high degree of variability. The GRAS domain is mainly composed of five motifs at the carboxyl (C-) terminal, leucine-rich region I, VHIID, leucine-rich region II, PFYRE, and SAW [19,20]. Notably, these five motifs play important roles in GRAS’s interactions with other proteins [21]. Because of this, the GRAS proteins are widely involved in many key processes of signal transduction, root radial elongation, axillary meristem formation, and stress response [22,23]. To date, GRAS gene families have been identified and analyzed in more than 30 monocotyledons and dicotyledons, including wheat and rose [24,25]. Previously, the *Arabidopsis* GRAS gene family was categorized into eight subfamilies, namely DELLA, SCL3, LAS, SCR, SHR, HAM, LISCL, and PAT1, based on conserved domains and functions [26,27]. The DELLA family contains the *GAI*, *RGA*, and *RGL* genes, which serve as major inhibitors of gibberellin signal transduction [28]. The SCL3 protein has been verified as a switch that mediates root elongation [29]. It has been reported that the LAS protein is closely related to lateral bud formation in the vegetative growth stage of *Arabidopsis* [30]. Additionally, during plant root growth, the SHR and SCR proteins tend to form SCR/SHR complexes [31]. Research indicates that in shoot apical meristem tissues, a group of key regulatory factors known as the Hairy Meristem (HAM) family GRAS domain proteins play a crucial role in determining the initiation and proliferation of shoot stem cells [32]. Moreover, the overexpression of *VaPAT1* (a *GRAS* gene of *Vitis amurensis*) improved the abiotic stress tolerance of transgenic *Arabidopsis* [33]. With the rapid development of sequencing technology, several new families, such as DLT, SCL4/7, Os19, Os4, and PT20, have gradually enriched the original GRAS genes subfamily [34].

Melatonin (N-acetyl-5-methoxytryptamine) is commonly found in plants, with roles that include acting as a plant biostimulant against biological and abiotic stress, promoting plant growth, and regulating the process of plant nutritional development [35]. Generally, the presence of melatonin can alleviate cold, heat, salinity, drought, ultraviolet radiation, and chemical toxicity [36,37]. Some results have indicated that treatment with 100 μmol L^−1^ melatonin significantly enhanced the growth of lettuce. Moreover, under high-temperature conditions, melatonin treatment was found to delay the bolting of lettuce [4]. Temperature plays a crucial role in the growth of plants; however, when the outside temperature reaches a certain level (30 °C), lettuce experiences heat stress. Heat stress (HS) is defined as a temperature increase that exceeds a threshold level and permanently affects plant growth and development [38]. It can disrupt the homeostasis of normal cells, leading to growth and developmental arrest, and even death. While members of the GRAS gene family play an important role in helping plants resist heat stress, there has been relatively little research on the relationship between heat stress and the GRAS gene families in lettuce.

In this study, we identified 59 *LsGRAS* gene members from the lettuce genome and identified their phylogenetic relationships, gene structure, motif composition, and chromosomal location. To investigate the expression profiles of these genes in lettuce, we analyzed the transcription levels of all 59 *LsGRAS* genes in the publicly available RNA-seq data under the high-temperature treatment conducted in the presence of exogenous melatonin. In addition, we studied the gene expression profiles of *LsGRAS* members in lettuce under high-temperature stress and analyzed cis elements in the promoter region of the *LsGRAS* gene. The *LsGRAS13* gene was screened and its function was analyzed. In conclusion, this study provides valuable insights into the role of the LsGRAS gene family in lettuce stress resistance, laying the groundwork for future research in this area.

## 2. Results

### 2.1. Identification of LsGRAS Genes in Lettuce

**Figure 1:**
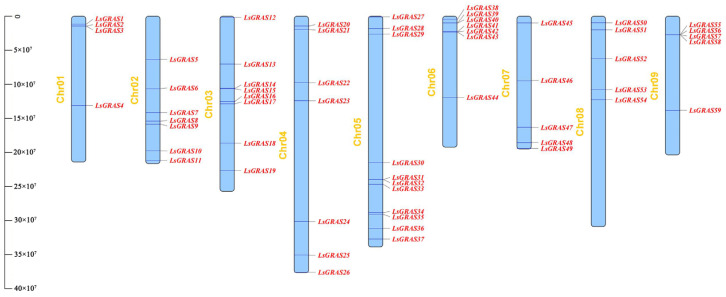
Distribution of *LsGRAS* genes on chromosomes of lettuce. The y-axis represents chromosome length.

In this study, 59 *LsGRAS* genes from lettuce were identified (Table 1). These genes were mapped at different locations on nine chromosomes (Figure 1). Based on their chromosomal locations, the 59 *LsGRAS* genes were named *LsGRAS1*–*LsGRAS59*. At the same time, some basic characteristics of the LsGRAS family members were analyzed, including protein length, isoelectric point (PI), protein molecular weight (MW), and predicted subcellular localization. The smallest protein was LsGRAS18, with 420 amino acids (aas), while the largest protein was LsGRAS34 (802 aa). Their molecular weights varied from 47,516.82 to 87,207.15 Dalton (Da). The pI values ranged from 4.9 to 8.6 (LsGRAS1 and LsGRAS6). All the LsGRAS proteins showed 100% nuclear localization based on the predicted subcellular localization (Table 1). The results of this study are important for understanding the gene regulatory network of lettuce and the growth and development processes of the plant. Analysis of the basic characteristics of the LsGRAS gene family provides more genetic resources for this study.

The distribution and interrelationships of plant genes are of great significance for revealing gene function and evolution. The results showed that the 59 *LsGRAS* genes were distributed across nine chromosomes. Among these chromosomes, there was a high number of *LsGRAS* genes on chromosome five, reaching 11, whereas the number of *LsGRAS* genes on chromosome one was the least, with only 4 (Figure 1). These results indicate that the distribution of *LsGRAS* genes on the chromosomes is uneven. Interestingly, 17 genes were adjacent to at least 1 other *LsGRAS* gene. For example, *LsGRAS2* and *LsGRAS3*, *LsGRAS14* and *LsGRAS15*, *LsGRAS31* and *LsGRAS32*, *LsGRAS34* and *LsGRAS35*, *LsGRAS39* and *LsGRAS40*, *LsGRAS41* and *LsGRAS43*, and *LsGRAS55* and *LsGRAS58* were adjacent to each other. These adjacent *LsGRAS* genes accounted for approximately 34% of the total number of genes.

### 2.2. Phylogenetic Analyses and Classifications of LsGRAS

**Figure 2:**
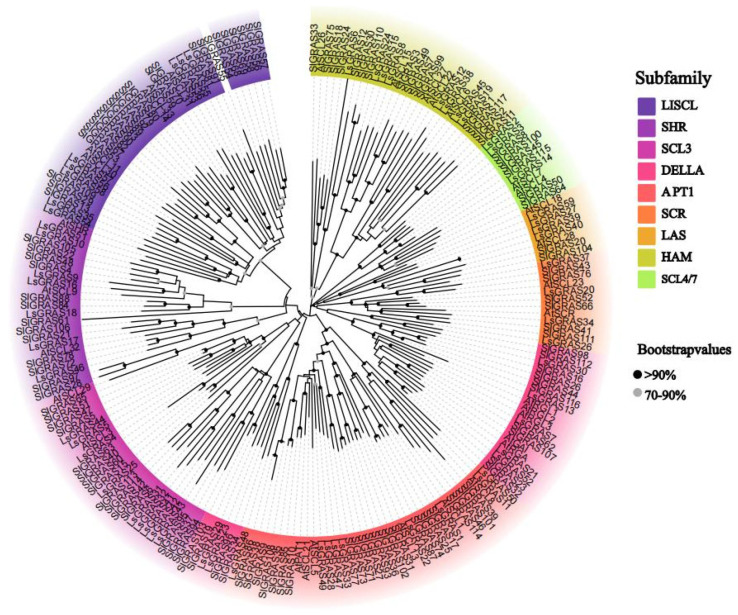
Phylogenetic tree of GRAS proteins in lettuce (LsGRAS), *Arabidopsis* (AtGRAS), and *Solanum lycopersicum* (SlGRAS). The phylogenetic tree is divided into nine distinct subfamilies, each represented by color, and all LsGRAS proteins are highlighted by their corresponding subfamily color.

In this study, 59 types of lettuce LsGRAS proteins were identified and constructed into a phylogenetic tree along with the known GRAS proteins of *Arabidopsis* and tomato (Figure 2). Based on the structure of the phylogenetic tree, the LsGRAS proteins were classified into nine main subfamilies. These subfamilies are DELLA, HAM, LAS, LISCL, PAT1, SCL3, SCL4/7, SCR, and SHR. Among them, the DELLA subfamily has seven members: LsGRAS7, LsGRAS13, LsGRAS21, LsGRAS23, LsGRAS29, LsGRAS30, and LsGRAS52. There are eight members of the HAM subfamily: LsGRAS6, LsGRAS8, LsGRAS12, LsGRAS15, LsGRAS19, LsGRAS24, LsGRAS38, and LsGRAS45. There are four members of the LAS subfamily: LsGRAS27, LsGRAS39, LsGRAS40, and LsGRAS59. There are eight members of the LISCL subfamily: LsGRAS35, LsGRAS36, LsGRAS53, LsGRAS54, LsGRAS55, LsGRAS56, LsGRAS57, and LsGRAS58. There are nine members of the PAT1 subfamily: LsGRAS1, LsGRAS11, LsGRAS22, LsGRAS28, LsGRAS33, LsGRAS47, LsGRAS48, LsGRAS49, and LsGRAS50. There are nine members of the SCL3 subfamily: LsGRAS4, LsGRAS5, LsGRAS25, LsGRAS31, LsGRAS32, LsGRAS41, LsGRAS42, LsGRAS43. and LsGRAS44. There are three members of the SCL4/7 subfamily: LsGRAS2, LsGRAS3, and LsGRAS14. There are four members of the SCR subfamily: LsGRAS20, LsGRAS26, LsGRAS34, and LsGRAS37. There are seven members of the SHR subfamily: LsGRAS9, LsGRAS10, LsGRAS16, LsGRAS17, LsGRAS18, LsGRAS46, and LsGRAS51. Importantly, there are adjacent LsGRAS members on different chromosomes, such as LsGRAS39 and LsGRAS40, LsGRAS35 and LsGRAS36, LsGRAS53 to LsGRAS58, LsGRAS47 to LsGRAS50, and LsGRAS4 with LsGRAS5. Additionally, pairs like LsGRAS31 and LsGRAS32, LsGRAS41 to LsGRAS44, LsGRAS2 and LsGRAS3, LsGRAS9 and LsGRAS10, and LsGRAS16 to LsGRAS18 indicate that tandem duplication is the primary evolutionary factor contributing to the amplification of LsGRAS.

### 2.3. Conserved Motifs and Proteins Structure of LsGRAS

**Figure 3:**
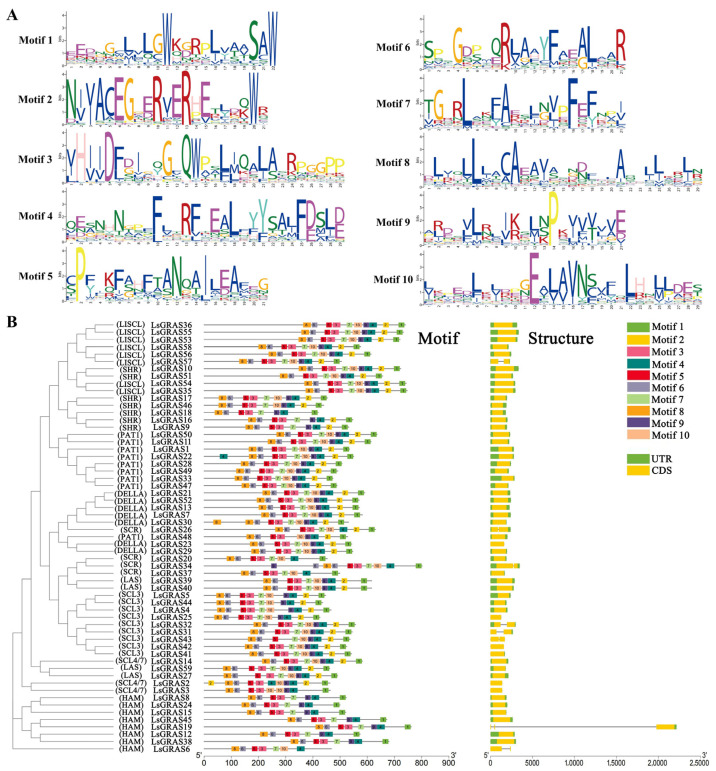
Phylogenetic clustering and gene structure of LsGRAS members. (**A**) Ten motif patterns of LsGRAS members are described. (**B**) On the left, the motif distribution of LsGRAS members is represented. Right: The green boxes represent the untranslated 5′ and 3′ regions, the yellow boxes represent exons, and the black lines represent introns.

To better understand the structure and characteristics of the LsGRAS proteins, we conducted a series of studies. First, a structural diagram was constructed using the LsGRAS motif scanning results to further display the structure of the LsGRAS proteins (Figure 3A). Surprisingly, we identified ten predicted LsGRAS protein conserved motifs and found that these motifs were the same across the 59 LsGRAS members from the DELLA subfamily, although the order of the motifs does not all appear to be in the same order. The genetic structures of these members were compared to further investigate the evolutionary lineage of the LsGRAS proteins. We observed that phylogenetically, the LsGRAS proteins members had similar exon numbers, lengths, and compositions. These findings indicate that the evolutionary lineage of the LsGRAS proteins is closely related to their genetic structure. This revealed the structure of the *LsGRAS* gene (Figure 3B). We found that the *LsGRAS gene* usually consists of one to three exons, with most of the genes (fifty-one) containing one exon. These results reveal the structure and genetic characteristics of the LsGRAS proteins and provide clues for further understanding their function and evolution.

### 2.4. Evolutionary Analyses of LsGRAS Gene Family Members

**Figure 4:**
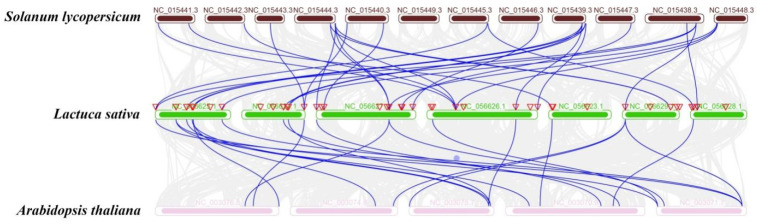
Evolutionary analyses of GRAS gene family members. Gray lines in the background indicate the collinear blocks within *Arabidopsis* and *Solanum lycopersicum* and lettuce, while blue lines highlight the syntenic *GRAS* gene pairs.

With these two representative species we constructed a composite map (Figure 4). We also analyzed the synthetic relationships among the 59 *LsGRAS* gene members in lettuce, *Arabidopsis*, and *Solanum lycopersicum* and found that 22 *LsGRAS* gene members were isogenic with *Arabidopsis* and 30 were isogenic with *Solanum lycopersicum* (Table 2). Therefore, the GRAS gene family plays a consistent role in different plants, indicating its significant contribution to plant evolution. Simultaneously, we constructed a composite map to enhance our understanding of the role of the LsGRAS gene family in the growth and development of lettuce, as well as its unique relationships with other species. It is worth noting that an isogenic relationship refers to a gene having the same function and sequence across different species. This finding also provides a basis for subsequent studies of related molecular mechanisms.

### 2.5. Transcriptome Analysis after Exogenous Melatonin Treatment under High-Temperature Conditions

**Figure 5:**
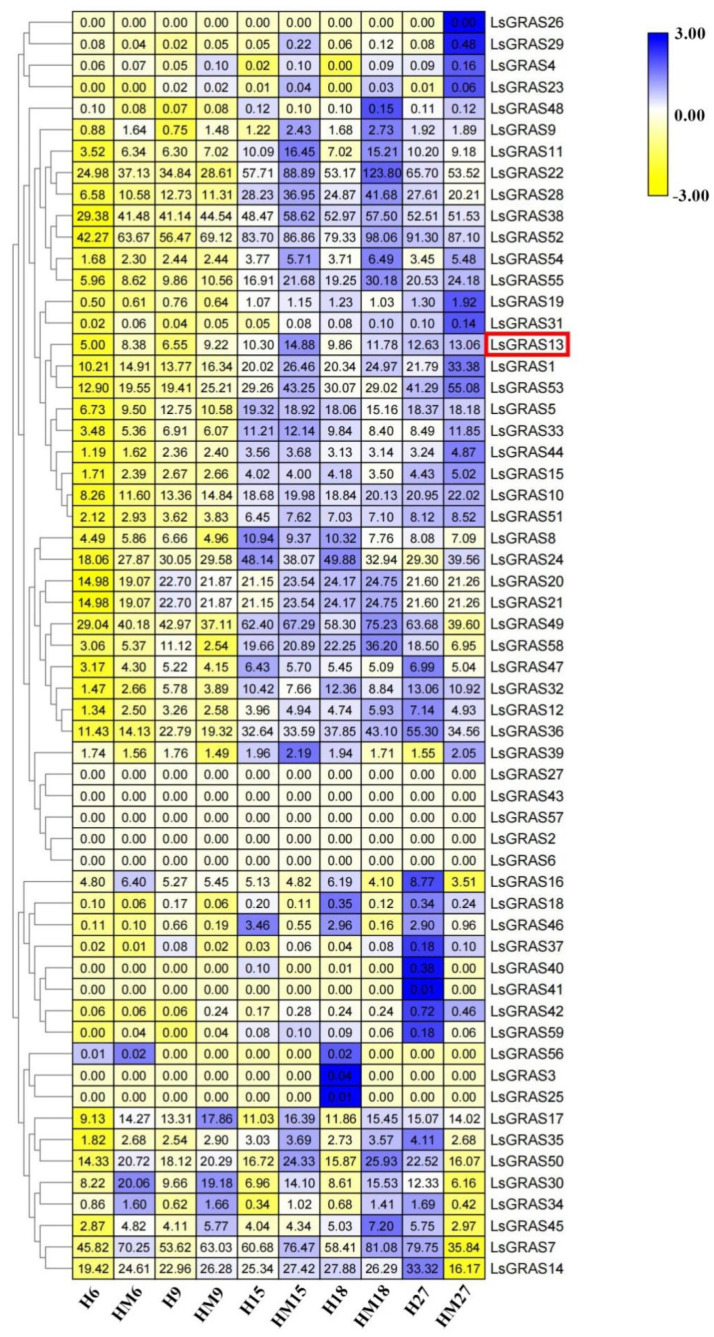
Gene expression profiles of *LsGRAS* members in lettuce. The leaves of plants at 6, 9, 15, 18, and 27 days were selected to analyze the expression profiles of *LsGRAS* members. ‘HM’ represents 100 μmol L^−1^ melatonin treatment at high temperature (35/30 °C), and ‘H’ represents no exogenous melatonin treatment at high temperature (35/30 °C). The target genes selected are highlighted in the red frame. As indicated in the legend, blue represents positive correlation and yellow represents negative correlation. The number in each cell signifies the degree of correlation, with a higher number indicating a stronger correlation.

To investigate the expression profiles of the 59 LsGRAS members in lettuce under high-temperature treatment, we conducted RNA-Seq analysis using publicly available leaf transcriptome data from NCBI (Bio project PRJNA810911). The results indicate that the expression levels of the *GRAS* genes in lettuce treated with exogenous melatonin at different time points were higher than those in treatments without exogenous melatonin under high-temperature conditions. This suggests that members of the GRAS family may play an essential role in melatonin-mediated heat stress resistance (Figure 5). The results reveal that the expression level of the *LsGRAS13* gene after high-temperature melatonin treatment (HM) was higher than that after without exogenous melatonin treatment (H) on days 6, 9, 15, 18, and 27. The expression trend showed a strong correlation with the growth and development of lettuce, peaking on day 15, followed by a gradual decline in growth. Additionally, the expression of *LsGRAS52* was the highest. Five *LsGRAS* genes were not expressed (*LsGRAS2, LsGRAS6, LsGRAS26, LsGRAS27, LsGRAS43, LsGRAS57*). Therefore, the change in the expression level of the *LsGRAS13* gene is proportional to the growth time, providing a more reliable reference for studying lettuce bolting compared to other irregular changes in genes.

Then, we conducted cis-component analysis (Table 3). These cis components include GA-responsive elements (P-box, TATC-box), light-responsive elements (G-box, CTt-Motif, MRE), auxin-responsive elements (Box 4), MeJA-responsive elements (CGTCA-motif), and salicylic-responsive elements (TCA) [27]. For instance, in this study, the gene was activated during high-temperature stress with exogenous melatonin, suggesting its role in regulating plant growth and development. This study provides important clues that further reveal the function of the *LsGRAS13* gene and its role in plant stress response and presents valuable information for genetic engineering and agricultural breeding research.

### 2.6. Silencing LsGRAS13 through VIGS Delays Lettuce Bolting

**Figure 6:**
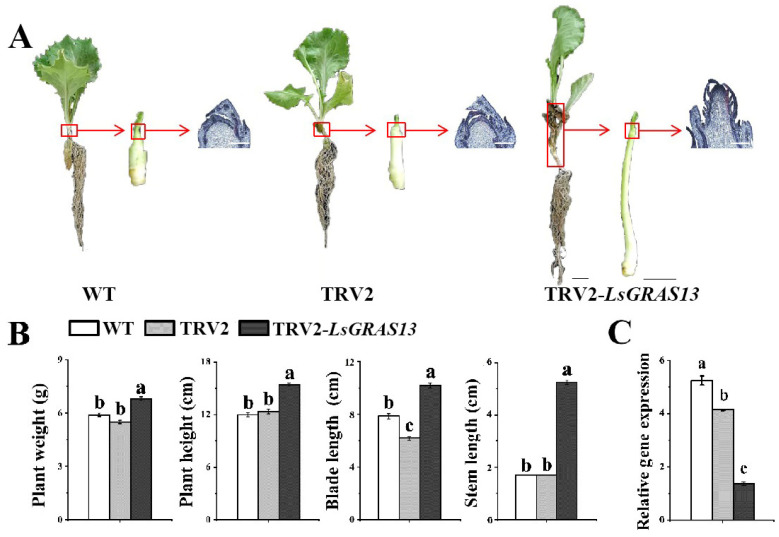
Phenotype of the *LsGRAS13* gene-silenced lettuce plants. (**A**) The bolting phenotype developed seven days after TRV2-*LsGRAS13* infection; scale bars = 1 cm. The paraffin section phenotype is also shown; scale bars = 100 μm. (**B**) The plant weight, plant height, blade length, and stem length of lettuce with TRV2-*LsGRAS13* treatments. (**C**) Utilizing the qRT-PCR method, we detected the relative expression levels of the *LsGRAS13* gene in lettuce plants. One-way analysis of variance (ANOVA) followed by Tukey’s multiple ange test was used to determine the values with significant variation (*p* < 0.05), which are indicated by different letters placed above the bars.

To explore the function of the *LsGRAS13* gene, we conducted transient transformation in lettuce. The results show that after two weeks of transient infection, the stem length of the TRV2-*LsGRAS13* plants increased, whereas no change was observed in the control group (Figure 6A,B). To observe the growth of lettuce stem tips, the stem tips of WT, TRV2, and TRV2-*LsGRAS13* plants were paraffin-selected, and it was found that the flower bud differentiation of pTRV2-*LsGRAS13* was more significant than that of the control (Figure 6A). As the buds differentiate, the stems undergo rapid expansion, causing the plant to grow taller, a phenomenon known as bolting. Finally, qRT-PCR detected a significant downregulation of over threefold in the relative expression level of *LsGRAS13* in the experimental plants compared to the control plants (Figure 6C). These findings suggest that the VIGS technique effectively silenced the *LsGRAS13* gene in lettuce and significantly increased the stem length of lettuce. The results show that *LsGRAS13* plays an important role in bolting lettuce and acts as a negative regulator of bolting.

## 3. Discussion

*GRAS* genes have been identified in various plant species, including 32 in *Arabidopsis* [21], 54 in *Solanum lycopersicum* [22], 117 in *Glycine max* [27], and 51 in *Medicago sativa* [14]. In this study, extensive analysis of the lettuce genome identified 59 putative *LsGRAS* genes (Table 1). These results were higher than those of *Arabidopsis* and *Solanum lycopersicum*, indicating that the LsGRAS gene family was amplified during lettuce evolution. Further studies have shown that the *LsGRAS* genes were distributed on nine chromosomes of lettuce, and approximately 34% of the genes were adjacent to each other (Figure 1), suggesting that tandem repetition is an important factor in GRAS gene family amplification [31]. These results suggest that there may be functional or regulatory relationships between the *LsGRAS* genes on chromosomes. On the basis of the discovery of its distribution and adjacency, we propose hypotheses to guide further research. For example, the increased number of *LsGRAS* genes on chromosome 5 may be associated with the significance of this chromosome in plant growth and development, and adjacent *LsGRAS* genes may share common regulatory factors or functional modules to coordinate plant physiological responses through interactions. In addition, by comparing the expression patterns and functional characteristics of the *LsGRAS* genes on other chromosomes, we could further reveal their diversity and evolutionary relationships. The genome size of lettuce (2099.84 Mb) was much higher than that of *Arabidopsis* (120.087 Mb) and *Solanum lycopersicum* (793.815 Mb) considering the differences in genome size, suggesting that the differences in the number of genes might be related to the genome size or characteristics of different species. The results of this study are of great significance for further understanding the evolutionary process, growth, and developmental regulation mechanisms of lettuce and its related agronomic applications.

Phylogenetic analysis is a common method used in the study of plant evolution, which helps to uncover the genetic relationships and evolutionary history of different species. In this study, the phylogenetic analysis of LsGRAS proteins revealed that they can be divided into nine subfamilies (Figure 2). LsGRAS members within the same subfamily or branch might have similar functions. This indicates that there is a correlation between structural similarity and functional similarity. In addition, we downloaded known *Solanum lycopersicum* genes from the database and compared them with lettuce data. However, *SlGRAS55* from tomato did not find a corresponding match, likely indicating the absence of a similar gene in lettuce; thus, it seems to be exclusive to *Solanum lycopersicum*. In conclusion, the structural consistencies and differences among the LsGRAS members can directly or indirectly reflect similarities and differences in their functions. Through phylogenetic analysis and genetic structure research, we explored the evolutionary process of plant genes.

It is important to analyze the evolutionary relationships of genes among different species to reveal the common characteristics and differences among species. This study indicates a high degree of homology among GRAS members in dicotyledonous plants [30]. In this study, the GRAS members of lettuce were analyzed and compared with those of *Arabidopsis* and *Solanum lycopersicum*. Surprisingly, the results are consistent with previous research showing that lettuce and *Solanum lycopersicum* have the highest homology, indicating that they may have retained some common features and functions through evolution. However, in *Arabidopsis*, the linear relationship between lettuce and *Arabidopsis* GRAS members was weak. This suggests that GRAS members may have undergone some special changes and adaptations during plant evolution and that such differences may be caused by evolutionary differences between species. It is noteworthy that we identified 22 and 30 homologous *LsGRAS* genes members between *Arabidopsis* and *Solanum lycopersicum* GRAS members, respectively (Table 3). This suggests that these orthologous relationships are conserved and likely existed before ancestral differentiation [27]. In conclusion, we speculated that the common characteristics of GRAS members in different species may be closely related to their evolutionary differences. Further exploration of the intersection of GRAS members in different species is of great interest for our understanding of the function and evolution of GRAS members.

In addition, transcriptome data and cis-acting element analysis can be used to understand the regulatory mechanism of the *GRAS* genes in stress treatment and phytohormone response, which is of great significance for further understanding the function and regulatory mechanism of the GRAS gene family [39]. By analyzing the transcriptome data and 59 LsGRAS proteins, we found that the change in the expression level of the *LsGRAS13* gene is proportional to the growth time, providing a more reliable reference for studying lettuce bolting compared to other irregular changes in genes (Figure 5). Furthermore, the analysis of cis-acting elements revealed the presence of numerous light-related and hormone-related cis-acting elements in the *LsGRAS13* gene (Table 3). P-box is an important component of the GA pathway that binds to TFs and plays a role in the response to GA-mediated osmotic stress signals [40]. DELLA proteins have shown potential importance in their association with AtGAI, AtRGA, and AtRGL1 in lettuce and other plants. Previous studies have shown that DELLA proteins are a branch of the GRAS gene family and play a negative regulatory role in GA signaling as possible transcriptional regulators [41]. Overall, the structural analysis provides clues about the subgroups to which GRAS members belong and reveals functional similarities between members within the same subfamily. In *Arabidopsis*, silencing the *RGL1* gene results in plants exhibiting delayed flowering and bolting [42].

## 4. Materials and Methods

### 4.1. Identification of LsGRAS

We downloaded the lettuce genome and genome annotation files from the Phytozome v12.1.6 database (https://phytozome.jgi.doe.gov/pz/portal.html, accessed on 3 February 2023); the CDS and protein sequences of the GRAS genes were extracted using TBtools [43,44,45]; and homology retrieval was performed using NCBI RefSeq and BLASTp [46,47]. To further identify the GRAS transcription factor family, the hidden Markov model (HMM) profile of the GRAS domain (PF03514) was retrieved from the Pfam database [48]. To confirm conserved domains among GRAS family members, we utilized HMMER SEARCH3.0 with a cutoff E value ≤ 0.01 using the SMART database.

To ensure the accuracy of the results, the BLAST-conserved domain search method was employed after manually removing duplicate protein sequences to search and identify the protein sequences within the domain. The ExPASy tool was used to predict the molecular weight and isoelectric point of the protein sequence, which are important for understanding the properties and functions of the protein [49]. Finally, WOLF PSORT was used to predict the subcellular localization of the LsGRAS proteins [50].

### 4.2. Phylogenetic Analyses and Classifications of the LsGRAS Proteins

The GRAS protein sequences of *Arabidopsis*, lettuce, and *Solanum lycopersicum* were compared using MEGA software (version 7.0; https://www.megasoftware.net/, accessed on 3 February 2023), while the Poisson model was used to estimate the evolutionary distance of the sequences, and the missing data processing method was used to deal with the missing data in the aligned sequences. To evaluate the reliability of the analysis results, 1000× Bootstrap resampling was performed. The GRAS phylogenetic relationships of *Arabidopsis* and lettuce were obtained by constructing phylogenetic trees of the GRAS proteins of *Arabidopsis* and lettuce using neighborhood linkage (NJ) [50]. To present the results of this study, phylogenetic tree images were modified and optimized using FigTree v1.4.3 (http://tree.bio.ed.ac.uk/software/figtree/, accessed on 4 February 2023) and Adobe Illustrator 2019 CC software to ensure clarity and readability.

### 4.3. Analysis of the LsGRAS Structure and Conserved Motifs, Gene Structure, and Phylogenetic Tree

This study utilized TBtools to describe the gene structure of the *GRAS* genes from the GFF3 file. MEME v5.1.1 (http://meme-suite.org/tools/meme, accessed on 4 February 2023) was employed to analyze the motifs of GRAS proteins [51], and MEGA was used for phylogenetic analysis [52].

### 4.4. LsGRAS Gene Chromosomal Locations, Duplications, and Synteny Analyses

Using genomic information sourced from Phytozome, the analysis determined the distribution of *LsGRAS* genes across each chromosome. Concurrently, the study examined the repetitive patterns of the *LsGRAS* genes, generating a repetition map to unveil gene duplication events within the lettuce genome. To explore the synergistic relationship between the homologous *LsGRAS* genes in lettuce and those in other species, we downloaded genomic data and the gene annotation files of *Arabidopsis* (TAIR annotation release 10) and *Solanum lycopersicum* (V1.1). The TBtools software was employed to create and construct synchronous analysis diagrams [51,52].

### 4.5. Transcriptome Analysis of Exogenous Melatonin Treated at High Temperature

We analyzed the transcriptome data of the *LsGRAS* genes in lettuce under high-temperature stress. When lettuce is cultivated for 20 days and the seedlings reach the five-leaf central stage, they are subjected to a high temperature of 35 °C/30 °C, with day and night variations, for a duration of 30 days. After the commencement of the heat treatment, a solution of 0 μmol L^−1^ melatonin (H) and a solution of 100 μmol L^−1^ melatonin (HM) were sprayed on the plants every morning at 9:00 am using a sprayer. Exogenous melatonin was applied every 3 days, and the plants were sampled at around 9 am after 30 days of high-temperature treatment. We selected leaves on days 0, 6, 9, 15, 18, and 27 (in triplicate), measured the physiological parameters of the leaves, and conducted RNA-seq analysis [4]. By analyzing the transcriptome data, we identified transcriptional changes associated with the *LsGRAS* genes under these conditions.

### 4.6. Cis-Element Analyses of LsGRAS13 Gene

The sequence located 2000 bp upstream of the transcription start site of *LsGRAS13* was selected and submitted to the PlantCARE website for predicting gene promoter regions [52]. The identification process was based on previous research recommendations for promoter region length and aimed to capture potential regulatory elements [16]. By submitting this sequence to PlantCARE, the type, location, and number of cis elements in the *LsGRAS13* gene sequence were determined and their functions in gene regulation were inferred.

### 4.7. Construction of the LsGRAS13 Gene Silencing Vector

The HiScript^®^IIQ RT SuperMix for qPCR (+gDNA wiper) vazyme kit (Vazyme, Piscataway, NJ, United States) was utilized with a 50 µL reaction system to complete three biological and three technical replicates for each sample. The *LsGRAS13* gene was cloned from lettuce cDNA, subjected to restriction through double enzyme digestion, and its fragment was amplified by using PCR. Simultaneously, the TRV2 vector (35 s promoter) underwent double enzyme digestion with EcoRI (GATTCTGTGAGTAAGGTTACCG) and BamHI (GAGACGCGTGAGCTCGGTACCG). The PCR primers used were *LsGRAS13*-F: CTTCGGATTGGAGGTCTGATGTGG and *LsGRAS13*-R: TGAACAGAATCGGAGGCAAGTTGAG. The recovered PCR product was then ligated with the vector to produce the recombinant plasmid. The identified recombinant plasmid was then transformed into Agrobacterium GV3101, and the infection solution was prepared. The infection buffer comprised 10 mM magnesium chloride, 10 mM MES buffer, and 20 mM acetylsyringone (MMA) [4]. The experiment was categorized into three groups: a blank control group (WT), a negative control group (TRV2-TRV1), and an experimental group (TRV2-*LsGRAS13*).

### 4.8. Infection of Lettuce Plants

The lettuce cultivar S39 was selected and cultivated indoors for 30 days in a pot filled with soil at a constant temperature of 23 °C ± 2. Subsequently, the experimental treatment was divided into the following groups: WT, where plants were not injected; TRV2, where an empty TRV2 and TRV1 vector were mixed in a 1:1 ratio and injected into plant leaves; and TRV2-*LsGRAS13*, where empty carriers of TRV2-*LsGRAS13* and TRV1 were mixed in a 1:1 ratio. After infection, it continued to grow for a week at 35/30 °C, while other growth conditions remain unchanged [4]. The qRT-PCR primers used were q*LsGRAS13*-F: TGGATCTTCGGATTGGAGGTCTG and q*LsGRAS13*-R: AACAACTCATCATCGCCACCATC.

### 4.9. Paraffin Sectioning Analyses

One week after infection, lettuce samples were selected. Approximately 1 cm of the stem tip was excised with a scalpel and then fixed in a prepared RNA-free FAA solution (composed of 38% formaldehyde 5 mL, glacial acetic acid 5 mL, and 50% ethanol 90 mL) for 24 h. After dehydration with various concentrations of alcohol, the material was immersed in a 1:1 xylene–ethanol solution for 0.5 h, followed by pure xylene for an additional 0.5 h. Next, we added finely chopped paraffin fragments to the xylene–ethanol solution until saturated and the material was then placed in a 57 °C thermostat overnight. The following day, xylene was evaporated in a constant temperature box at 60 °C for 1 h, the stock liquid was discarded, and the pure wax was changed twice within 4.5 h. The wax-saturated stem tip and paraffin wax were poured into a paper box, cooled at room temperature, and solidified into wax blocks. The wax block was trimmed and sliced using a microtome. The slices were spread on a clean slide, and absorbent paper was used to remove excess water. At room temperature, the water evaporated and the slices were placed in a constant temperature box at 37 °C overnight for drying [3]. Finally, following a conventional method, the processes included dewaxing, rehydration, dyeing, dehydration, until transparency was achieved for observation and photography.

## 5. Conclusions

We identified 59 LsGRAS genes in lettuce that were unevenly distributed across nine chromosomes. All the LsGRAS proteins demonstrated 100% nuclear localization based on the predicted subcellular localization and were phylogenetically categorized into nine conserved subfamilies. Our investigation highlighted the involvement of the *LsGRAS13* gene in lettuce growth and development. The functional studies revealed that silencing the *LsGRAS13* gene accelerated the bolting process in lettuce. The paraffin section results indicated a significantly faster flower bud differentiation rate in *LSGRAS13*-silenced plants compared to control plants. In summary, through systematic exploration and preliminary characterization of the GRAS gene family in lettuce, we have taken the initial steps toward understanding this gene family. However, further research is essential to unveiling the role of the LsGRAS genes in other biological processes and to exploring the functions and regulatory mechanisms of the entire gene family.

## Figures and Tables

**Table 1:** Detailed information of *LsGRAS*.

Gene ID	mRNA ID	Protein ID	Gene Name	Length	PI	MW	SL
LOC111904158	XM_023899939.2	XP_023755707.1	*LsGRAS1*	535	4.9	60,331.9	Nucleus
LOC111916701	XM_023912371.1	XP_023768139.1	*LsGRAS2*	463	8.6	53,145	Nucleus
LOC111916700	XM_023912370.2	XP_023768138.1	*LsGRAS3*	464	6.31	52,883.18	Nucleus
LOC111921793	XM_023917371.2	XP_023773139.1	*LsGRAS4*	467	5.67	52,701.63	Nucleus
LOC111875951	XM_023872476.2	XP_023728244.1	*LsGRAS5*	447	6.05	50,251.7	Nucleus
LOC111905160	XM_023900832.1	XP_023756600.1	*LsGRAS6*	468	4.9	54,276.87	Nucleus
LOC111883408	XM_023879739.2	XP_023735507.1	*LsGRAS7*	582	5.02	63,282.65	Nucleus
LOC111883461	XM_023879795.2	XP_023735563.1	*LsGRAS8*	523	5.41	58,477.35	Nucleus
LOC111900213	XM_023896081.2	XP_023751849.1	*LsGRAS9*	532	5.64	60,046.82	Nucleus
LOC111905757	XM_023901493.2	XP_023757261.1	*LsGRAS10*	723	5.95	81,370.2	Nucleus
LOC111888457	XM_023884628.2	XP_023740396.1	*LsGRAS11*	614	6.51	67,805.15	Nucleus
LOC111892896	XM_023888978.2	XP_023757261.1	*LsGRAS12*	574	6.08	63,627.1	Nucleus
LOC111920919	XM_023916486.2	XP_023772254.1	*LsGRAS13*	571	5.27	62,495.69	Nucleus
LOC111885810	XM_023882048.2	XP_023737816.1	*LsGRAS14*	582	4.94	64,787.71	Nucleus
LOC111919376	XM_023914966.2	XP_023770734.1	*LsGRAS15*	521	5.44	57,716.52	Nucleus
LOC111903110	XM_023898897.2	XP_023754665.1	*LsGRAS16*	547	5.91	61,708.32	Nucleus
LOC111880266	XM_023876680.2	XP_023732448.1	*LsGRAS17*	454	5.7	50,433.69	Nucleus
LOC111898333	XM_023894263.2	XP_023750031.1	*LsGRAS18*	420	5.16	47,516.82	Nucleus
LOC111884683	XM_042900756.1	XP_042756690.1	*LsGRAS19*	762	5.93	85,207.15	Nucleus
LOC111879947	XM_023876376.2	XP_023732144.1	*LsGRAS20*	453	5.52	49,702.44	Nucleus
LOC111890374	XM_023886497.2	XP_023742265.1	*LsGRAS21*	591	5.12	63,529.61	Nucleus
LOC111886167	XM_023882398.2	XP_023738166.1	*LsGRAS22*	550	5.81	59,325.03	Nucleus
LOC111921515	XM_023917098.2	XP_023772866.1	*LsGRAS23*	543	7.6	61,064.99	Nucleus
LOC111901852	XM_023897710.2	XP_023753478.1	*LsGRAS24*	500	5.12	56,090.62	Nucleus
LOC111885807	XM_023882047.1	XP_023737815.1	*LsGRAS25*	426	6.95	48,433.9	Nucleus
LOC111921756	XM_023917341.2	XP_023773109.1	*LsGRAS26*	630	5.33	70,435.78	Nucleus
LOC111888709	XM_023884834.2	XP_023740602.1	*LsGRAS27*	491	8.34	56,173.88	Nucleus
LOC111914874	XM_023910585.2	XP_023766353.1	*LsGRAS28*	507	5.49	56,480.36	Nucleus
LOC111880523	XM_023876955.2	XP_023732723.1	*LsGRAS29*	547	6.72	61,357.13	Nucleus
LOC111897910	XM_023893864.2	XP_023749632.1	*LsGRAS30*	531	5.16	58,419.36	Nucleus
LOC111878348	XM_023874860.2	XP_023730628.1	*LsGRAS31*	545	5.26	61,012.01	Nucleus
LOC111878163	XM_023874675.2	XP_023730443.1	*LsGRAS32*	557	5.05	62,755.68	Nucleus
LOC111918681	XM_023914310.2	XP_023770078.1	*LsGRAS33*	474	6.25	53,731.38	Nucleus
LOC111891717	XM_023887787.2	XP_023743555.1	*LsGRAS34*	802	6.15	55,501.91	Nucleus
LOC111890649	XM_023886757.2	XP_023742525.1	*LsGRAS35*	747	6.04	84,426.2	Nucleus
LOC111907281	XM_023903060.2	XP_023758828.1	*LsGRAS36*	740	5.2	83,659.51	Nucleus
LOC111891561	XM_023887613.2	XP_023743381.1	*LsGRAS37*	499	6.15	55,501.91	Nucleus
LOC111886881	XM_023883122.2	XP_023738890.1	*LsGRAS38*	680	6.07	74,775.27	Nucleus
LOC111900413	XM_023896302.2	XP_023752070.1	*LsGRAS39*	616	5.64	60,046.82	Nucleus
LOC111900414	XM_023896303.2	XP_023752071.1	*LsGRAS40*	616	5.88	68,694.99	Nucleus
LOC111890431	XM_023886550.2	XP_023742318.1	*LsGRAS41*	542	5.65	60,825.06	Nucleus
LOC111890433	XM_023886553.1	XP_023742321.1	*LsGRAS42*	525	5.07	59,047.92	Nucleus
LOC111895192	XM_042896049.1	XP_042751983.1	*LsGRAS43*	537	5.63	60,602.17	Nucleus
LOC111880474	XM_023876909.2	XP_023732677.1	*LsGRAS44*	436	6.57	49,181.56	Nucleus
LOC111881387	XM_023877780.2	XP_023733548.1	*LsGRAS45*	672	5.89	74,919.83	Nucleus
LOC111898450	XM_023894356.2	XP_023750124.1	*LsGRAS46*	440	5.59	49,587.6	Nucleus
LOC111902851	XM_023898665.2	XP_023754433.1	*LsGRAS47*	489	6.94	55,144.69	Nucleus
LOC111884953	XM_023881251.2	XP_023737019.1	*LsGRAS48*	528	5.01	59,119.49	Nucleus
LOC111896695	XM_023892671.2	XP_023748439.1	*LsGRAS49*	492	5.09	54,974.1	Nucleus
LOC111908450	XM_023904280.2	XP_023760048.1	*LsGRAS50*	636	6.52	68,912.59	Nucleus
LOC111893308	XM_023889368.2	XP_023745136.1	*LsGRAS51*	657	6.38	74,389.54	Nucleus
LOC111881444	XM_023877838.2	XP_023733606.1	*LsGRAS52*	570	4.98	62,044.82	Nucleus
LOC111880342	XM_023876767.2	XP_023732535.1	*LsGRAS53*	721	5.38	81,983.71	Nucleus
LOC111917088	XM_023912753.2	XP_023768521.1	*LsGRAS54*	743	7.59	84,084.93	Nucleus
LOC111902221	XM_023898073.2	XP_023753841.1	*LsGRAS55*	733	5.15	83,122.89	Nucleus
LOC111902222	XM_023898074.2	XP_023753842.1	*LsGRAS56*	614	7.79	69,694.81	Nucleus
LOC111902234	XM_023898086.2	XP_023753854.1	*LsGRAS57*	505	7.12	58,006.6	Nucleus
LOC111902223	XM_023898076.2	XP_023753844.1	*LsGRAS58*	575	7.19	66,079.86	Nucleus
LOC111897993	XM_023893945.2	XP_023749713.1	*LsGRAS59*	462	6.48	53,238.42	Nucleus

Note. Gene ID: accession number of lettuce locus ID; mRNA ID: mRNA expression sequence; Protein IS: protein identification; Gene Name: gene named for its position on the chromosomes; Length: protein length in amino acids; PI: isoelectric points; MW: molecular weight in Daltons; SL: subcellular localization.

**Table 2:** Screening for homologous *LsGRAS* genes in lettuce, *Arabidopsis,* and *Solanum lycopersicum*.

*Lactuca sativa*	*Arabidopsis thaliana*	*Lactuca sativa*	*Solanum lycopersicum*
LOC111920919	NM_101361.3	LOC111884683	NM_001346910.1
LOC111892896	NM_130079.3	LOC111892896	NM_001346910.1
LOC111920919	NM_126218.3	LOC111885810	LOC101257128
LOC111884683	NM_130079.3	LOC111903110	LOC101262675
LOC111880266	NM_114855.4	LOC111880266	LOC101257522
LOC111919376	NM_119835.6	LOC111892896	NM_001346907.1
LOC111903110	NM_119928.3	LOC111900213	LOC101262675
LOC111880266	NM_001037090.2	LOC111883461	LOC101251641
LOC111883408	NM_105306.4	LOC111888457	NM_001247398.1
LOC111900213	NM_119928.3	LOC111888457	LOC100037501
LOC111888457	NM_124630.5	LOC111883408	NM_001247436.1
LOC111888709	NM_104434.4	LOC111880523	LOC101264916
LOC111918681	NM_126521.3	LOC111918681	LOC101252411
LOC111918681	NM_124189.6	LOC111888709	NM_001247250.1
LOC111890374	NM_126218.3	LOC111918681	LOC101253917
LOC111901852	NM_119835.6	LOC111891561	LOC101248329
LOC111904158	NM_101996.4	LOC111891717	LOC101255150
LOC111881387	NM_130079.3	LOC111918681	NM_001247376.2
LOC111881387	NM_130079.3	LOC111921756	LOC101261388
LOC111881387	NM_115927.5	LOC111901852	LOC101251641
LOC111881387	NM_116232.5	LOC111886167	LOC101260037
LOC111900413	NM_104988.2	LOC111886167	LOC101247427
		LOC111886167	LOC101253917
		LOC111886167	NM_001247376.2
		LOC111904158	NM_001306164.1
		LOC111881387	NM_001346910.1
		LOC111902851	LOC101253917
		LOC111902851	LOC101252411
		LOC111890431	LOC101244899
		LOC111900413	LOC101244695

**Table 3:** The cis-component analysis of the *LsGRAS13* promoter.

Site Name	Matrix Sequence	Position	Strand	Motif Annotation
G-box	CACGAC	796	+	cis-acting regulatory element involved in light responsiveness
G-box	CACGAC	1052	+	cis-acting regulatory element involved in light responsiveness
Circadian	CAAAGATATC	1078	−	cis-acting regulatory element involved in circadian control
P-box	CCTTTTG	1352	+	gibberellin-responsive element
TCA-element	CCATCTTTTT	1320	+	cis-acting element involved in salicylic acid responsiveness
TGA-box	TGACGTAA	1669	−	part of an auxin-responsive element
Box 4	ATTAAT	804	+	part of a conserved DNA module involved in light responsiveness
TCT-motif	TCTTAC	1519	−	part of a light-responsive element
MSA-like	(T/C)C(T/C)AACGG(T/C)(T/C)A	1192	+	cis-acting element involved in cell cycle regulation
CGTCA-motif	CGTCA	113	+	cis-acting regulatory element involved in MeJA responsiveness
CGTCA-motif	CGTCA	1672	+	cis-acting regulatory element involved in MeJA responsiveness
CGTCA-motif	CGTCA	1964	+	cis-acting regulatory element involved in MeJA responsiveness
TATC-box	TATCCCA	1529	+	cis-acting element involved in gibberellin responsiveness
GT1-motif	GGTTAAT	1976	+	light-responsive element
MRE	AACCTAA	314	−	MYB binding site involved in light responsiveness

## Data Availability

The datasets generated and/or analyzed during the current study are available in the NCBI repository, Bio project PRJNA810911.

## References

[1] Chen Z., Han Y., Ning K., Ding Y., Zhao W., Yan S., Luo C., Jiang X., Ge D., Zhang X. (2017). Inflorescence Development and the Role of *LsFT* in Regulating Bolting in Lettuce (*Lactuca sativa* L.). Front. Plant Sci..

[2] Fukuda M., Matsuo S., Kikuchi K., Kawazu Y., Fujiyama R., Honda I. (2011). Isolation and functional characterization of the *FLOWERING LOCUS T* homolog, the *LsFT* gene, in lettuce. J. Plant Physiol.

[3] Wang L., Wu Y., Du W., Yan Z., Qi Z., Tang W., Han Y., Liu C., Fan S., Hao J. (2021). Virus-induced gene silencing (VIGS) analysis shows involvement of the *LsSTPK* gene in lettuce (*Lactuca sativa* L.) in high temperature-induced bolting. Plant Signal. Behav..

[4] Chen L., Xu M., Liu C., Hao J., Fan S., Han Y. (2022). *LsMYB15* regulates bolting in leaf lettuce (*Lactuca sativa* L.) under high-temperature stress. Front. Plant Sci..

[5] Han R., Truco M.J., Lavelle D.O., Michelmore R.W. (2021). A composite analysis of flowering time regulation in lettuce. Front. Plant Sci..

[6] Zheng Y., Luo L., Gao Z., Liu Y., Chen Q., Kong X., Yang Y. (2019). Grafting induces flowering time and tuber formation changes in *Brassica* species involving *FT* signalling. Plant Biol..

[7] Huot B., Yao J., Montgomery B.L., He S.Y. (2014). Growth-defense tradeoffs in plants: A balancing act to optimize fitness. Mol. Plant.

[8] Liu R., Su Z., Zhou H., Huang Q., Fan S., Liu C., Han Y. (2020). LsHSP70 is induced by high temperature to interact with calmodulin, leading to higher bolting resistance in lettuce. Sci. Rep..

[9] Du P., Wu Q., Liu Y., Cao X., Yi W., Jiao T., Hu M., Huang Y. (2022). WRKY transcription factor family in lettuce plant (*Lactuca sativa*): Genome-wide characterization, chromosome location, phylogeny structures, and expression patterns. PeerJ.

[10] Park J.S., Kim J.B., Cho K.J., Cheon C.I., Sung M.K., Choung M.G., Roh K.H. (2008). *Arabidopsis* R2R3-MYB transcription factor AtMYB60 functions as a transcriptional repressor of anthocyanin biosynthesis in lettuce (*Lactuca sativa*). Plant Cell Rep..

[11] Zhang B., Tong Y., Luo K., Zhai Z., Liu X., Shi Z., Zhang D., Li D. (2021). Identification of growth-regulating factor transcription factors in lettuce (*Lactuca sativa*) genome and functional analysis of *LsaGRF5* in leaf size regulation. BMC Plant Biol..

[12] Ning K., Han Y., Chen Z., Luo C., Wang S., Zhang W., Li L., Zhang X., Fan S., Wang Q. (2019). Genome-wide analysis of MADS-box family genes during flower development in lettuce. Plant Cell Environ..

[13] Liu M., Huang L., Ma Z., Sun W., Wu Q., Tang Z., Bu T., Li C., Chen H. (2019). Genome-wide identification, expression analysis and functional study of the GRAS gene family in Tartary buckwheat (*Fagopyrum tataricum*). BMC Plant Biol..

[14] Yang D.L., Yao J., Mei C.S., Tong X.H., Zeng L.J., Li Q., Xiao L.T., Sun T.P., Li J., Deng X.W. (2012). Plant hormone jasmonate prioritizes defense over growth by interfering with gibberellin signaling cascade. Proc. Natl. Acad. Sci. USA.

[15] Wen C.K., Chang C. (2002). *Arabidopsis RGL1* encodes a negative regulator of gibberellin responses. Plant Cell.

[16] Li M., Sun B., Xie F., Gong R., Luo Y., Zhang F., Yan Z., Tang H. (2019). Identification of the GRAS gene family in the *Brassica juncea* genome provides insight into its role in stem swelling in stem mustard. PeerJ.

[17] Sun X., Jones W.T., Rikkerink E.H. (2012). GRAS proteins: The versatile roles of intrinsically disordered proteins in plant signalling. Biochem. J..

[18] Khan Y., Xiong Z., Zhang H., Liu S., Yaseen T., Hui T. (2022). Expression and roles of GRAS gene family in plant growth, signal transduction, biotic and abiotic stress resistance and symbiosis formation-a review. Plant Biol..

[19] Li S., Zhao Y., Zhao Z., Wu X., Sun L., Liu Q., Wu Y. (2016). Crystal structure of the GRAS domain of SCARECROW-LIKE7 in *Oryza sativa*. Plant Cell.

[20] Sun X., Xie Z., Zhang C., Mu Q., Wu W., Wang B., Fang J. (2016). A characterization of grapevine of GRAS domain transcription factor gene family. Funct. Integr. Genom..

[21] Zeng X., Ling H., Chen X., Guo S. (2019). Genome-wide identification, phylogeny and function analysis of GRAS gene family in *Dendrobium catenatum* (*Orchidaceae*). Gene.

[22] Niu Y., Zhao T., Xu X., Li J. (2017). Genome-wide identification and characterization of GRAS transcription factors in tomato (*Solanum lycopersicum*). PeerJ.

[23] Jaiswal V., Kakkar M., Kumari P., Zinta G., Gahlaut V., Kumar S. (2022). Multifaceted roles of GRAS transcription factors in growth and stress responses in plants. iScience.

[24] To V.T., Shi Q., Zhang Y., Shi J., Shen C., Zhang D., Cai W. (2020). Genome-wide analysis of the GRAS gene family in barley (*Hordeum vulgare* L.). Genes.

[25] Kumari P., Gahlaut V., Kaur E., Singh S., Kumar S., Jaiswal V. (2023). Genome-wide identification of GRAS transcription factors and their potential roles in growth and development of rose (*Rosa chinensis*). J. Plant Growth Regul..

[26] Liu Y., Wang W. (2021). Characterization of the GRAS gene family reveals their contribution to the high adaptability of wheat. PeerJ..

[27] Wang L., Ding X., Gao Y., Yang S. (2020). Genome-wide identification and characterization of GRAS genes in soybean (*Glycine max*). BMC Plant Biol..

[28] Tyler L., Thomas S.G., Hu J., Dill A., Alonso J.M., Ecker J.R., Sun T.P. (2004). Della proteins and gibberellin-regulated seed germination and floral development in *Arabidopsis*. Plant Physiol..

[29] Heo J.O., Chang K.S., Kim I.A., Lee M.H., Lee S.A., Song S.K., Lee M.M., Lim J. (2011). Funneling of gibberellin signaling by the GRAS transcription regulator scarecrow-like 3 in the *Arabidopsis* root. Proc. Natl. Acad. Sci. USA.

[30] Greb T., Clarenz O., Schafer E., Muller D., Herrero R., Schmitz G., Theres K. (2003). Molecular analysis of the LATERAL SUPPRESSOR gene in *Arabidopsis* reveals a conserved control mechanism for axillary meristem formation. Genes Dev..

[31] Zhang B., Liu J., Yang Z.E., Chen E.Y., Zhang C.J., Zhang X.Y., Li F.G. (2018). Genome-wide analysis of GRAS transcription factor gene family in *Gossypium hirsutum* L.. BMC Genom..

[32] Geng Y., Zhou Y. (2021). HAM gene family and shoot meristem development. Front. Plant Sci..

[33] Yuan Y., Fang L., Karungo S.K., Zhang L., Gao Y., Li S., Xin H. (2016). Overexpression of *VaPAT1*, a GRAS transcription factor from *Vitis amurensis*, confers abiotic stress tolerance in *Arabidopsis*. Plant Cell Rep..

[34] Tian C., Wan P., Sun S., Li J., Chen M. (2004). Genome-wide analysis of the GRAS gene family in rice and *Arabidopsis*. Plant Mol. Biol..

[35] Arnao M.B., Hernández-Ruiz J. (2018). Melatonin and its relationship to plant hormones. Ann. Bot..

[36] Erdal S. (2019). Melatonin promotes plant growth by maintaining integration and coordination between carbon and nitrogen metabolisms. Plant Cell Rep..

[37] Altaf M.A., Shahid R., Ren M.X., Mora-Poblete F., Arnao M.B., Naz S., Anwar M., Altaf M.M., Shahid S., Shakoor A. (2021). Phytomelatonin: An overview of the importance and mediating functions of melatonin against environmental stresses. Physiol. Plant.

[38] Perrella G., Bäurle I., van Zanten M. (2022). Epigenetic regulation of thermomorphogenesis and heat stress tolerance. New Phytol..

[39] Wang T., Liu M., Wu Y., Tian Y., Han Y., Liu C., Hao J., Fan S. (2022). Genome-wide identification and expression analysis of MAPK gene family in lettuce (*Lactuca sativa* L.) and functional analysis of *LsMAPK4* in high- temperature-induced bolting. Int. J. Mol. Sci..

[40] Yi X., Gao H., Yang Y., Yang S., Luo L., Yu C., Wang J., Cheng T., Pan H. (2021). Differentially expressed genes related to flowering transition between once- and continuous-flowering roses. Biomolecules.

[41] Waseem M., Nkurikiyimfura O., Niyitanga S., Jakada B.H., Shaheen I., Aslam M.M. (2022). GRAS transcription factors emerging regulator in plants growth, development, and multiple stresses. Mol. Biol. Rep..

[42] Xu J., Kong L., Ren W., Wang Z., Tang L., Wu W., Liu X., Ma W., Zhang S. (2024). Identification and expression analysis of TPS family gene in *Cannabis sativa* L.. Heliyon.

[43] Büyük İ., İlhan E., Şener D., Özsoy A.U., Aras S. (2019). Genome-wide identification of CAMTA gene family members in *Phaseolus vulgaris* L. and their expression profiling during salt stress. Mol. Biol. Rep..

[44] Gomez M.D., Barro-Trastoy D., Fuster-Almunia C., Tornero P., Alonso J.M., Perez-Amador M.A. (2020). Gibberellin-mediated RGA-LIKE1 degradation regulates embryo sac development in *Arabidopsis*. Journal of Experimental Botany.

[45] Lv G., Zheng X., Duan Y., Wen Y., Zeng B., Ai M., He B. (2021). The GRAS gene family in watermelons: Identification, characterization and expression analysis of different tissues and root-knot nematode infestations. PeerJ.

[46] Chen C., Chen H., Zhang Y., Thomas H.R., Frank M.H., He Y., Xia R. (2020). TBtools-an integrative toolkit developed for interactive analyses of big biological data. Mol. Plant.

[47] Chang J., Fan D., Lan S., Cheng S., Chen S., Lin Y., Cao S. (2023). Genome-wide identification, expression and stress analysis of the GRAS gene family in *Phoebe Bournei*. Plants.

[48] Duvaud S., Gabella C., Lisacek F., Stockinger H., Ioannidis V., Durinx C. (2021). Expasy, the Swiss Bioinformatics Resource Portal, as designed by its users. Nucleic Acids Res..

[49] Horton P., Park K.J., Obayashi T., Fujita N., Harada H., Adams-Collier C.J., Nakai K. (2007). WoLF PSORT: Protein localization predictor. Nucleic Acids Res..

[50] Bailey T.L., Williams N., Misleh C., Li W.W. (2006). MEME: Discovering and analyzing DNA and protein sequence motifs. Nucleic Acids Res..

[51] Kumar S., Stecher G., Tamura K. (2016). MEGA7: Molecular Evolutionary Genetics Analysis Version 7.0 for Bigger Datasets. Mol. Biol. Evol..

[52] Lescot M., Déhais P., Thijs G., Marchal K., Moreau Y., Van de Peer Y., Rouzé P., Rombauts S. (2002). PlantCARE, a database of plant cis-acting regulatory elements and a portal to tools for in silico analysis of promoter sequences. Nucleic Acids Res..

